# New Composite Hydrogel Based on Whey and Gelatin Crosslinked with Copper Sulphate

**DOI:** 10.3390/ma15072611

**Published:** 2022-04-01

**Authors:** Pompilia Mioara Purcea Lopes, Dumitriţa Moldovan, Marioara Moldovan, Rahela Carpa, Codruţa Saroşi, Petru Păşcuţă, Amalia Mazilu Moldovan, Radu Fechete, Violeta Popescu

**Affiliations:** 1 Physics and Chemistry Department, Technical University of Cluj-Napoca, 28 Memorandumului Str., 400114 Cluj-Napoca, Romania; mioara.lopes@im.utcluj.ro (P.M.P.L.); dumitrita.moldovan@phys.utcluj.ro (D.M.); petru.pascuta@phys.utcluj.ro (P.P.); amalia.mazilu@gmail.com (A.M.M.); rfechete@phys.utcluj.ro (R.F.); 2 Polymeric Composite Laboratory, Institute of Chemistry Raluca Ripan, Babeş-Bolyai University, 30 Fȃntȃnele Str., 400294 Cluj-Napoca, Romania; marioara.moldovan@ubbcluj.ro (M.M.); liana.sarosi@ubbcluj.ro (C.S.); 3 Department of Molecular Biology and Biotechnology, Faculty of Biology and Geology, Babeș Bolyai University, M. Kogălniceanu Street, 400084 Cluj-Napoca, Romania; rahela.carpa@ubbcluj.ro

**Keywords:** whey protein isolate, composite hydrogels, swelling kinetics, crystallinity secondary and tertiary protein structure, NMR relaxometry, antibacterial properties

## Abstract

By-products from the meat and dairy industries are important sources of high biological value proteins. This paper explores possibilities for improving the swelling and integrity of a cross-linked whey and gelatin hydrogel with different amounts of CuSO_4_ × 5H_2_O. Overall, swelling tests demonstrate that cross-linked samples show a better hydration capacity and stability in the hydration medium, but different copper concentrations lead to different swelling behavior. At concentrations smaller than 0.39%, the sample lasts for 75 h in a water environment before beginning to disintegrate. At a concentration of copper sulphate higher than 0.55%, the stability of the sample increased substantially. The swelling kinetics has been investigated. The diffusion constant values increased with the increase in copper concentration, but, at the highest concentration of copper (0.86%), its value has decreased. Spectroscopy analyses such as Fourier transform infrared (FT-IR), X-ray diffraction (XRD), ultraviolet-visible spectroscopy (UV-VIS), and nuclear magnetic resonance (NMR) relaxometry analyses revealed changes in the secondary and tertiary structure of proteins as a result of the interaction of Cu^2+^ ions with functional groups of protein chains. In addition to its cross-linking ability, CuSO_4_ × 5H_2_O has also shown excellent antibacterial properties over common bacterial strains responsible for food spoilage. The result of this research demonstrates the potential of this hydrogel system as a unique material for food packaging.

## 1. Introduction

Protein derivatives are obtained by capitalizing on the by-products resulting from the processing of vegetable or animal origin raw materials rich in proteins. Whey is one of the main providers of protein derivatives and is used for the preparation of a wide range of hydrogels with applications in medicine [1,2], food and packaging industry [3,4,5,6], agriculture [7,8], environmental protection [9,10], and fuel production, such as hydrogen gas [11,12]. A large number of scientific research papers continue to confirm the physicochemical and functional properties along with the nutritional and economical values of whey proteins.

Globular whey proteins, represented by α-Lactalbumin (α-La) (about 23%), of which secondary structure consists of α-helixes and β-folds, β-Lactoglobulin (β-Lg) (74.1%) with β-folds and α-helix in their secondary structure [13] and serum bovine albumins (SBA) (around 1.6%). The protein that leads to the gelification process is β-Lg (α-Lactoglobulins do not polymerize at temperatures higher than 70 °C), through the hydrophilic functional groups as -OH, -CONH, -CONH_2_, -COOH, -SO_3_H and ionic groups [14,15], determining the mechanical properties of the hydrogel [16,17].

The formation of the three-dimensional structure that results in the gelation process of whey involves the following: conformational changes in the structure of native proteins; the formation of bisulfite bridges inside and between peptide chains through chemical reactions and hydrophobic interactions [16].

Despite its high qualities, the mechanical properties of whey hydrogels are limited; therefore, to improve these properties it is necessary to use specific compounds such as polymers, multivalent ions, essential oils, etc. [14,18]. Thus, polysaccharides such as xanthan gum, tragacanth, and pectin can be used to model the controlled release capabilities of whey-based hydrogels [19]; gellan gum and CaCl_2_ improve the ability of whey hydrogels to release the caffeine from the polymer network [14]; cellulose nanofibers, TiO_2_ nanoparticles and rosemary essential oil increase the hydrophobicity, elasticity, antibacterial and antioxidant properties of whey hydrogel [1]; k-carrageenan increases the density of a whey-based hydrogel and delays the release of curcumin during digestion [20]. Many researchers manifest great interest in copper (II) and its compounds as cross-linkers and, no less, as antimicrobial agents for composite hydrogels with various applications, such as medicine and food packaging [6,21,22]. Being a widespread metal in living systems and cheaper than gold and silver (two other metals with antibacterial properties), copper nanoparticles (CuNPs) are also suitable to be obtained by eco-friendly biosynthetic methods [23,24,25,26,27,28,29,30,31].

Regarding the characteristics of compounds added to the whey-based hydrogel, the hydrogels containing acidic tannins with different molecular weights and chemical structures demonstrate lower swelling ability, due to their hydrophobicity [16].

Gunasekaran et al. [15] explain the swelling process as a diffusion of the solvent into the pores of the hydrogel at the same time as a relaxation of the polymer network is followed by the solvent penetration.Therefore, the hydrogel suffers a plasticization and an increase in volume. The findings of their research work show that, based on pH values of the swelling medium, for the swelling degree up to 60%, at pH 10.00 the diffusion is of the first order (Fickian model) and the swelling equilibrium was reached in ~50 min; however, at pH 1.8 and pH 7.6 the swelling process follows an atypical diffusion, and the time for reaching the swelling equilibrium has doubled.

X-ray diffraction is a method of studying the properties of crystalline materials and the effect of their blending in whey hydrogels. The diffractograms of pure hydrogels show wide peaks with low intensity [18], as they have amorphous states, naturally with crystalline domains, due to the presence of ordered structures such as α-helixes from proteins. When they are filled with different crystalline substances, the last ones induce changes in the secondary structures of proteins and therefore in their diffractograms. So, the more homogenous the mixtures of crystalline and non-crystalline phases, the lower the intensity of their diffractograms peaks [1].

The spectroscopic analysis in the field of IR (400–4000 cm^−1^) makes a significant contribution to understanding the composition and structure of compounds in the food industry, especially their proteinaceous components [16,32,33]. The peptide bonds in the α-helical and β-sheet structures, the coil, and disorganized/random areas of the proteins can be estimated by analyzing the amide I bands (1600–1700 cm^−1^) that correspond with the secondary structure of proteins [34]. Nevertheless, the addition of certain chemical compounds (natural or synthetic polymers, cations, essential oils, plasticizers, or reinforcing agents) aims to improve the mechanical, physicochemical, and biological properties of whey hydrogels, which will contribute to the structural changes of proteins. This is because they show absorption peaks independent of the complexity of the composite hydrogel, causing shifts of absorption peaks to lower or higher wavenumbers or intensities, respectively, which will highlight possible new bonds formed in the structure of the hydrogel [33].

Porous media are largely characterized using a non-destructive method, such as nuclear magnetic resonance (NMR) relaxometry [34,35,36], a method that analyzes the molecular motions of water molecules inside the polymer matrix, in the millisecond range. Water plays an important role in hydrogels production, since it influences the mechanical and rheological characteristics of hydrogels, because of interactions between a fraction of water with the polymer chains; therefore, this method can describe the structural formation of hydrogels. Spin–spin ^1^H transverse relaxation times (*T*_2_) measurements using CPMG (Carr–Purcell–Meiboom–Gill) pulse sequence is commonly used as it is a rapid and robust method, while the longitudinal relaxation time (*T*_1_) measurements require more time [35].

Ozel et al. [36] have applied ^1^H spin-lattice relaxation data for quantitative determinations of water molecules in the polymer network of whey composite hydrogels with black carrot extract and to demonstrate the highly anisotropic translation diffusion also. NMR relaxometry by *T_2_* measurements provides information about the effect of polysaccharides (xanthan, pectin, alginate, and sunflower) on the swelling degree of whey-based composite hydrogel blended with xanthan, pectin, alginate, and sunflower [37]. Deng et al. [38] have developed a semi-dynamic gastric simulator to study the protein denaturation (hydrolysis) and pH values in the gastric segment of the digestive system with the NMR relaxometry via *T*_1_ and *T*_2_ measurements of free amino groups (NH_2_) and protein concentrations over time.

Our previous research [6] has opened up the prospect of studying a hydrogel system based on whey proteins, hydrolyzed whey proteins, gelatin, and copper with improved hydration properties. This research investigated the effect of copper sulphate concentration on the swelling properties of the hydrogels, to determine the minimum concentration of copper that assure the stability of hydrogels in water for several days. It is the first paper that studies the structural changes of whey/gelatin hydrogel as a function of copper sulphate concentration by X-ray diffraction and correlates the result with those obtained by NMR relaxometry.

## 2. Materials and Methods

### 2.1. Materials

Whey Protein Isolate (WPI) ISOLAC produced by Carbery Group Carbery (Cork, Ireland) and distributed by S.C. Way Better Nutrition from Cluj-Napoca, Gelatin Reagent (AMRESCO, LLC, Fountain Parkway Solon, Cleveland, OH, USA), glycerol (Sigma-Aldrich, Taufkirchen, Germany), and CuSO_4_ × 5H_2_O (Sigma-Aldrich, Taufkirchen, Germany) have been used for the obtaining of the hydrogels.

### 2.2. Preparation of Hydrogels

There were prepared five samples with the same amount of gelatin (g), glycerol solution (1:1 *w*/*w*), and whey (WPI) solution of 5 % (*w*/*w*). For an optimal dissolution and homogenization of the whey, the samples were mixed with a magnetic stirrer AREX Heating Magnetic stirrer (VELP SCIENTIFICA, Usmate, Italy) for 2 h at 800 rpm. Gelatin was added to previously heated water at 60 °C, followed by ultrasonication in degas mode to remove the formed foam. The samples were brought to the same weight by the addition of distilled water and 5% CuSO_4_ × 5H_2_O solutions in different proportions. The wet hydrogel samples contain 7.81% gelatin, 6.25% glycerol, 3.12% whey, and different amounts of copper sulphate, according to Table 1.

### 2.3. Swelling Degree

The swelling degree was determined by submerging the hydrogel into distilled water, and weighted at predetermined time intervals, up to a constant weight [6]. The dried and weighted samples (0.2–0.3 g) were introduced in pre-moistened tea bags, heat-sealed, and placed in a water swelling environment. In the first 2 h, the samples were removed from distilled water at 5 min intervals, the excess water was removed by swabbing with a paper towel then weighed. In the next step, samples were weighed at 15 min intervals for 5 h, then once a day for 8 days until reaching a constant weight.

The time-dependent swelling degree (*SW*%) was calculated with Equation (1) [6], considering the weight of the teabag (*W_1_*), the weight of dried hydrogel (*W_2_*), and the weight of the swelled hydrogel (*W_3_*):


(1) $$SW\;(\%)=\frac{W_{3}-W_{2}-W_{1}}{W_{2}}\cdot 100$$

For the evaluation of the kinetics of the swelling process, Equation (2) was used [15,18]:

(2) $$\frac{SW_{t}}{SW_{e}}=Kt^{n}$$

where *SW_t_* and *SW_e_* represent the weight of the hydrogel at time *t* and at equilibrium (*e*), respectively, *K* is the rate constant of the water intake through the hydrogel matrix and *n* is the diffusion exponent for the swelling process.

The characteristic constant *K* of the swelling process is dependent on the geometry of the hydrogel sample and the diffusion coefficient. Equation (2) is valid when *SW_t_/*S*W_e_* < 0.6.

Depending on the exponent *n*, this equation describes three developing ways of the sorption process [15,39] as follows:

- First order/First Fick’s Law (*n*~0.5) describes the perfect Fickian model of the swelling process (the slow diffusion of the solvent in the hydrogel matrix).
- Second-order Law (*n* = 1.0) explains that the mobility of the solvent is much higher than the degree of relaxation of the hydrogel matrix and the amount of solvent diffused is proportional to time.
- The atypical case of sorption (0.5 < *n* < 1.0) is when the solvent diffusion and the hydrogel matrix relaxation are comparable (the hydrogel matrix does not relax enough for the solvent to penetrate).

The linearized form of Equation (2) is:

(3) $$ln\left(\frac{SW_{t}}{SW_{e}}\right)=ln\left(K\right)+n\;ln\left(t\right)$$

The exponent *n* of the swelling process was calculated from the slope of the plot $ln\left(SW_{t}/SW_{e}\right)=f${*ln*$\left(t\right)$} for swelling degrees < 60% [15,18].

Another Equation (4) specific to Fickian diffusion that evaluates the swelling degree (*SW*) depending on the rate constant *K*, time *t,* and diffusion exponent *n* is the following:


(4) $$SW_{t}=K\cdot t^{n}$$

If there is a linear correspondence between *SW_t_* and *t*^1/2^ one can deduce that the diffusion process follows the Fickian model.

Swelling kinetics for the second-order diffusion was evaluated with Equation (5) [39].

(5) $$\frac{dSW}{dt}=K{\left(SW-SW_{e}\right)}^{2}$$

where *dSW/dt* is swelling rate, *K* is the rate constant (1/h), *SWe* is the swelling degree at equilibrium.

By integration between the limits *SW = 0* to *SW = SW_e_* and *t = 0* to *t*, Equation (5) leads to Schott’s Equation (6):

(6) $$\frac{t}{SW}=\frac{1}{KSW_{e}^{2}}+\frac{t}{SW_{e}}$$

Plotting *t/SW* as a function of *t*, a straight line with slope *1/SW_e_* and intercept of *1/K·SW_e_^2^* can be obtained. From the slope of the plot *t/SW* = f(*t*), the (theoretical) value for *SW_e_* and the rate constant *K* were calculated:

(7) $$SW_{e}=\frac{1}{slope}$$

and

(8) $$K=\frac{1}{Intercept\times SW_{e}^{2}}$$

### 2.4. X-ray Diffraction

The X-ray diffraction of the samples was performed with the XRD-6000 SHIMADZU (Kyoto, Japan) diffractometer with CuKα radiation (λ = 1.54184 Å) and the 2θ angle range between 10 and 60° [2,40,41].

For the identification of the crystalline phases in the analyzed samples, the powder diffraction file (PDF) was used.

For the calculation of space between diffraction planes, Bragg’s law was used [1,2,40]:

(9) $$d_{h,k,l}=\frac{n\lambda }{2sin\theta }\;(Å)$$

where *n* represents the diffraction order; *θ* is the Bragg angle, *λ* is the wavelength of the radiation used for the diffraction, and *h*, *k*, *l* represent the Miller indices of diffraction plan. The value of *n* is 1 for most of the cases.

### 2.5. FTIR Spectroscopy

Fourier transform infrared (FT-IR) spectroscopy of both precursors and hydrogels was performed using a Bruker Tensor 27 FT-IR Spectrometer (Bruker Optik GmbH, Ettlingen, Germany) in attenuated total reflection (ATR) mode [1,6].

### 2.6. UV-VIS Spectroscopy

UV-VIS spectra of the hydrogels have been measured on reflection mode using a double beam UV-VIS spectrometer (Jasco V-750) provided with an integrating sphere of 150 mm JASCO model ILV924, JASCO Corporation, Tokyo, Japan [6,42].

### 2.7. ^1^H NMR Relaxometry

The *T*_2_-distributions were obtained by Inverse Laplace Transform of the CPMG (Carr-Purcell-Meiboom-Gill) decays measured using a Bruker Minispec mq20 NMR spectrometer (Bruker, Karlsruhe, Germany) [43]. A number of 1000 echoes were recorded with 70 μs echo time and a recycle delay of 0.5 s. For a good signal-to-noise ratio, a number of 512 scans were recorded.

### 2.8. Antimicrobial Activity

Antimicrobial evaluation of the hydrogel samples was conducted for *Porphyromonas gingivalis* ATCC 33277, *Enterococcus faecalis* ATCC 29212, *Streptococcus mutans* ATCC 25175, *Escherichia coli* ATCC 25922, and *Staphylococcus aureus* ATCC 25923 from the Microbiology Laboratory collection of the Biology and Geology Faculty of UBB Cluj, by agar well diffusion method. After the incubation period (28 h) at a temperature of 27 °C (hydrogels samples melt above this temperature) were determined the zones of inhibition (mm) in the tested microbial strains.

Each bacterial strain was grown for 24 h on a nutrient agar-agar medium (Atlas, 2010). Then, a 0.5 McFarland dilution was made from each strain in sterile saline. From these dilutions, each Petri dish was inoculated with a sterile swab soaked in 0.5 McFarland microbial suspension streaked over the entire surface on the solid culture medium (Mueller Hinton-Oxoid) then left to dry for 20 min at 37 °C. The samples cut into 5 mm diameter discs were picked up with sterile tweezers and applied to the solid culture medium. Incubation was performed for 28 h at 27 °C (at higher temperatures the gels melted). Reading was conducted by measuring the diameter of the zone of inhibition: the larger the diameter of the zone of inhibition, the higher the sensitivity of the bacteria to the respective antibacterial substances [44,45].

## 3. Results and Discussions

### 3.1. Swelling Tests

Swelling is one of the most important qualities of whey hydrogels for the most common applications. It is a continuous process of polymer network penetration by the solvent, in which the hydrogel is introduced. A mobile front is created, which, as the pores of the polymer network are occupied by the solvent, expands, and allows the solvent to advance inside hydrogel [17,39].

The swelling degree of hydrogels depends on their composition, such as the proportion of constituents in the polymer matrix and the cross-linking degree. The hydrophilic functional groups on the whey and gelatin protein chains act synergistically in the swelling process.

**Figure 1:**
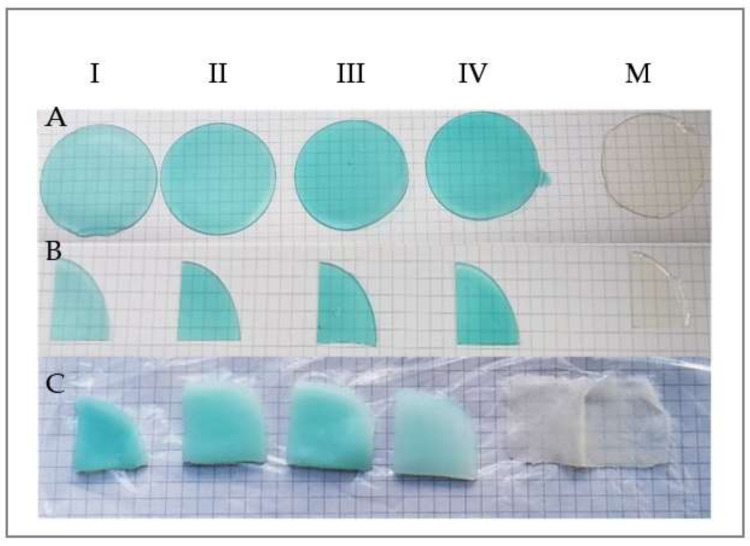
Hydrogels samples. (**A**) dried samples of hydrogels; (**B**) the weighed samples before swelling; (**C**) the samples after 8 days of swelling. M = control sample (0% CuSO_4_ × 5H_2_O), I = sample with 0.3906% CuSO_4_ × 5H_2_O, II = sample with 0.5469% CuSO_4_ × 5H_2_O, III = sample with 0.7031% CuSO_4_ × 5H_2_O, IV = sample with 0.8594 CuSO_4_ × 5H_2_O.

The samples of hydrogels before and after swelling are presented in Figure 1.

The cross-linked samples with CuSO_4_ × 5H_2_O kept their integrity even after 8 days of swelling; meanwhile, the control sample disintegrated completely, leaving just little traces on the teabag.

**Figure 2:**
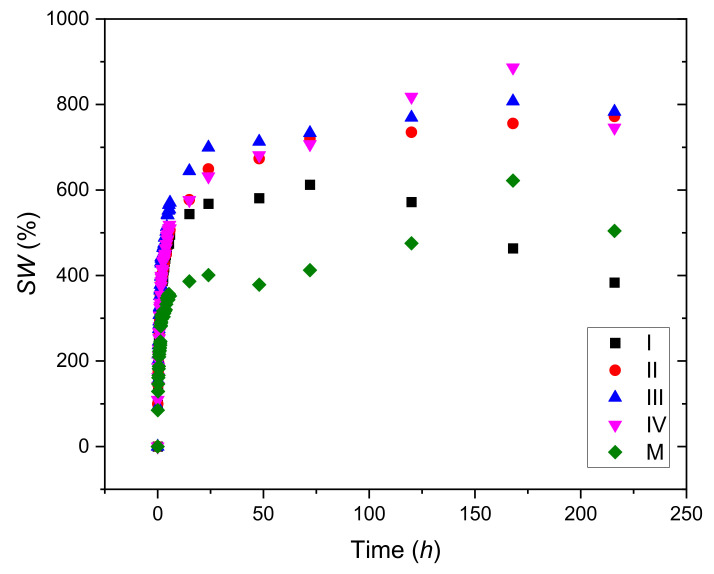
Swelling degree as a function of time for hydrogels samples with different concentrations of CuSO_4_ × 5H_2_O.

The results obtained for the swelling degree are presented in Figure 2.

One can observe that CuSO_4_ has a favorable effect on the degree of swelling. The hydrogels crosslinked with CuSO_4_ × 5H_2_O demonstrate a higher hydration capacity than the control sample (M), due to cross-linking effect of copper ions. Sample III, the one with 0.86% CuSO_4_ × 5H_2_O, stands out clearly compared to the other samples, starting from the very first 10 min of swelling, keeping its trend throughout the testing period.

Examining the plot from Figure 2, one can conclude that the minimum concentration of copper sulphate for the obtaining of long-lasting hydrogels is 0.55%. The increasing of the concentration of copper led to the increasing of the quantity of water uptake, due to the increasing of the stability of the hydrogel that had been crosslinked due to the presence of copper (II) ions.

Hydrogels containing copper swell very rapidly in the first 25–30 min of the process, and then, the quantity of water increased almost linearly with time up to 175 min in the case of samples containing 0.55–0.85% copper (samples (II, III and IV). Sample I, containing 0.39% copper sulphate, began to lose weight after 75 min because the number of ionic bonds formed by copper was too small.

A similar behavior related to water intake has been shown for hydrogels based on whey proteins at neutral pH by Sundaram Gunasekaran [15], who concluded that the hydrogels are sensitive to pH changes and the swelling rate is higher in an alkaline environment. In the case of hydrogels containing whey, the equilibrium has been established rapidly (50–100 min) as a function of pH; in the case of hydrogels containing whey, acrylamide, N,N′-methylenebisacrylamide, and carbopol, the swelling process continued up to 72 h at pH of 1.2 or 24 h at pH of 7.4 [18].

The presence of hydrophilic groups plays an important role in the gel-forming ability of whey proteins, while also improving the water sorption capacity of hydrogels. The enhanced swelling capacity of hydrogels at neutral pH is due to the presence of ionized carboxylate groups, resulting in repulsion between the carboxylate groups [18].

The whey proteins have an optimal ability to swell at neutral to slightly alkaline pH of the swelling environment: pH 7.4 [18], pH 7.6 [15], pH~6.5–8 [46]. At these pH values, the positive charges of the swelling medium neutralize most of the negative charges of the hydrogel network, which leads to a decrease in electrostatic rejections, the swelling being achieved due to the relaxation of the hydrogel network [15,18].

Mayorova et al. [16] combined the hydrophobic and antitumor properties of tannin acids existing in plants and fruits with whey, in different proportions, to obtain a hydrogel that was introduced to swell, in simulated digestive fluids with different pH values (5.0/7.0/9.0). The hydrogel demonstrated superior swelling abilities at pH 9.0 compared with the swelling at pH 5.0. So, the higher the pH, the higher the exposure of the electrostatic functional groups and the repulsive forces between them, and therefore the higher the swelling capacity of the hydrogel [16]. On the other hand, the acidic pH of swelling mediums (such as gastric fluids) reduces the swelling ability of whey hydrogels. As amphoteric polyelectrolytes, whey proteins have a minimum absorption capacity at their isoelectric point (pI = 5.1) [15,16,17], where the positive and negative charges on the peptide chains are in equilibrium. So, this is a characteristic that can be useful for the controlled release hydrogels in the digestive tract.

**Figure 3:**
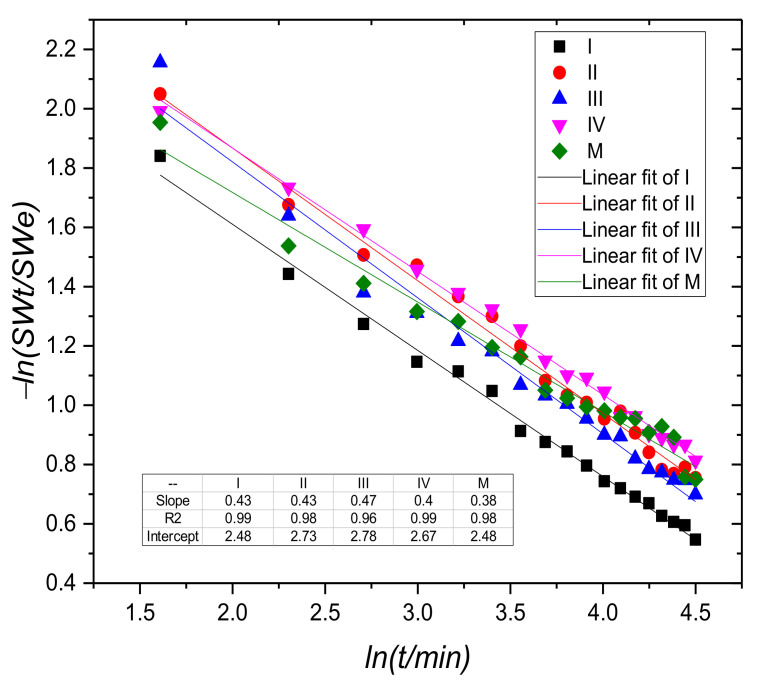
The plot of −*ln (SWt/SWe)* against *ln(t)* for samples containing copper and the control sample (M) for swelling degree < 60%.

The plot of *ln(SWt/SWe)* against *ln(t)* (Equation (3)) for samples containing copper and the control sample (M) for swelling degree <60% is presented in Figure 3. The swelling process of the hydrogel samples under study follows as can be seen from Figure 3, the Fickian model, the exponent *n* being close to the value 0.5 and R^2^ close to the value 1. In this case, the diffusion of the solvent is the rate-determining step, because the rate of solvent penetration is slower than the chain relaxation rate [15,47].

The diffusion constant K values are increasing with the increase in copper concentration in samples I, II, and III, but the value of the constant *K* for the sample with a minimum content of Cu^2+^ is close to that of the hydrogels without copper. One explanation could be that the degree of crosslinking was small. However, swelling experiments have shown that the integrity of swelled hydrogels is maintained even at low degrees of cross-linking.

**Figure 4:**
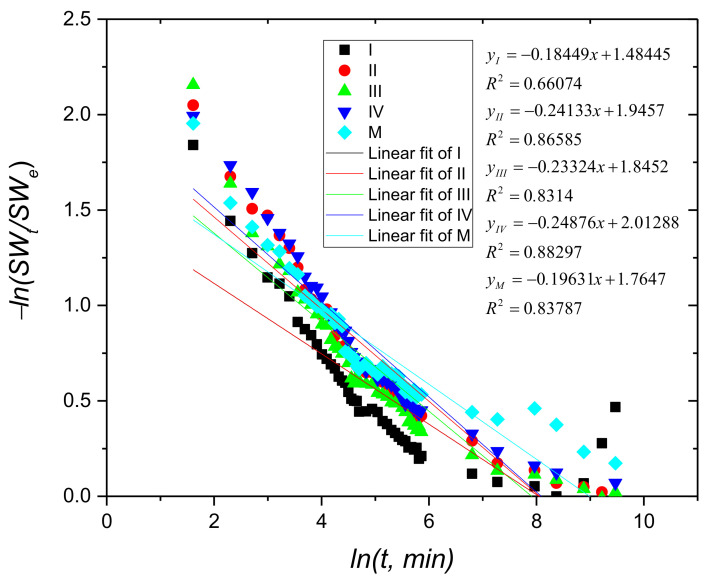
The plot of −*ln (SWt/SWe)* against *ln(t)* for samples containing copper and the control sample (M) for the whole swelling process.

As can be seen from Figure 4, the swelling process of whey-based hydrogels crosslinked with Cu^2+^, describes non-Fickian kinetics throughout whole swelling process (*R^2^* has no values close to 1), due to the numerous interactions between functional groups of the hydrogel matrix and water molecules [37,40,48].

**Figure 5:**
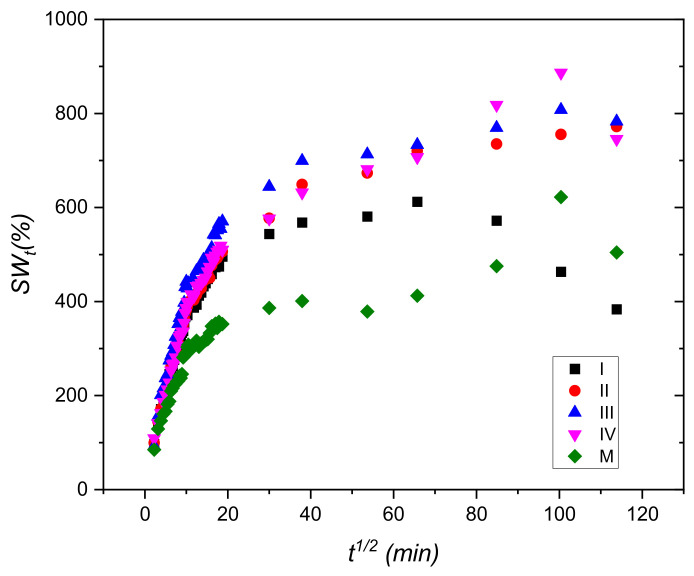
The dependence between *SW_t_* and *t*^1/2^.

Figure 5 illustrates the dependence between *SW_t_* and f(*t^1/2^)* on the whole swelling process. The plots not being linear, we confirm that the swelling does not follow the Fickian model.

**Figure 6:**
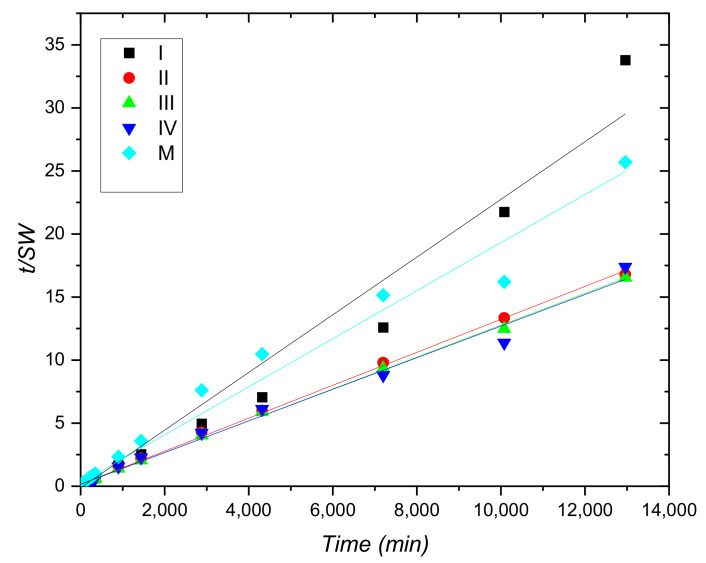
The plot *t/SW* as a function of *t*.

Representing Schott’s Equation (6), it can be observed in Figure 6, that, except for sample containing 0.39% copper sulphate, one can see a linear dependence between *t/SW* and *t*, demonstrating that the swelling process follows Schott’s equation for all swelling time.

The results obtained demonstrate a good correlation between the swelling degree experimentally determined and the one based on the kinetics of the swelling process (Table 2).

The authors obtained similar results in previous work on a hydrogel system of hydrolyzed whey, gelatin, and copper sulphate [6]. Schott’s second-order kinetics has also been described by other authors on different hydrogel systems, such as sodium alginate and acrylic acid hydrogel cross-linked by copolymerization with N, *N*-methylene-bis-(acrylamide) [49]. The formation of hydrogen bonds in the cross-linking process caused an increase in the rigidity of the hydrogel, which explains the higher mobility of the solvent compared to the degree of relaxation of the polymer matrix throughout the swelling process.

### 3.2. XRD Diffraction

**Figure 7:**
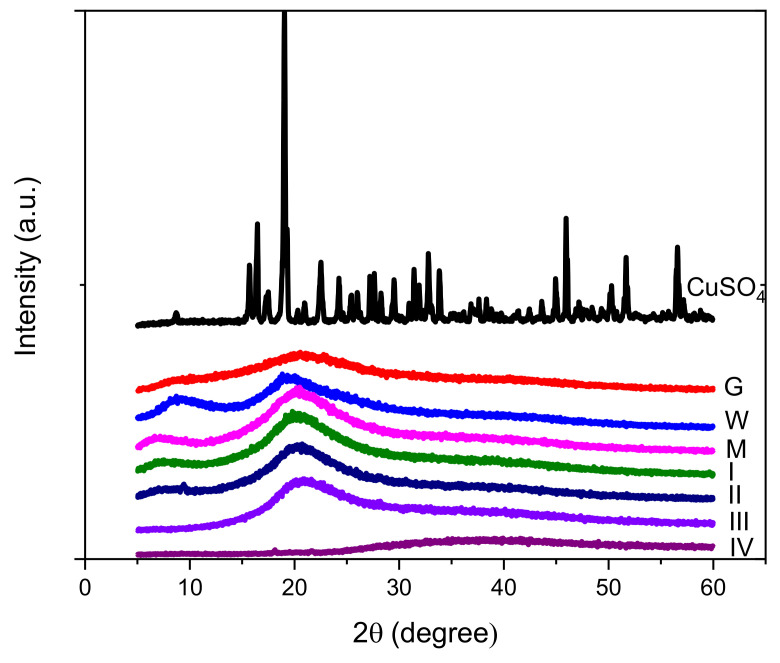
XRD patterns for copper sulphate, gelatin (G), whey powder (W), whey–gelatin hydrogel (M), and hydrogels containing copper sulphate (I, II, III, IV).

Figure 7 presents XRD patterns for copper sulphate, gelatin, whey powder, and hydrogels containing copper sulphate. The diffraction pattern of CuSO_4_ has been identified based on JCPDS 77-190. In the diffraction pattern of hydrogels containing copper, no diffraction peaks of CuSO_4_ were found, revealing the lack of the crystalline copper sulphate into the structure of the hydrogels.

Gelatin diffraction pattern exhibit two diffraction peaks, one located to 2θ = 9.89° and the other at 20.21°. These peaks found at smaller 2θ by other authors (around 7° and 19°) [2,40,48,50] or 8.1° [40]) are assigned to triple helix structure from gelatin. The peak from 2θ ≈ 8° corresponds to a distance of around 1.1 nm between each turn of the helical structure of gelatin [51] or the diameter of the triple helix [52,53].

The area of the peak around 2θ = 8° can be used for the estimation of the content of helix structures [41,48,52,53], while the peak at 2θ ≈ 20° is associated with the spacing between polypeptide chains [2]. The intensity of the peak at 2θ ≈ 9.9° is smaller for the powder of gelatin compared to the XRD patterns presented by other authors [51], revealing a low crystallinity of the gelatin powder used as raw material for hydrogel production [40].

The diffraction pattern of whey exhibit two characteristic peaks at 2θ ≈ 9.0° and 2θ ≈ 19.8°, while for the reference sample containing only whey and gelatin, the values for 2θ ≈ 7.1° and 2θ ≈ 20.4°. The interaction between gelatin and whey conducted to the formation of helical structures with higher distances between the turns, the value increased from 0.89 nm (corresponding to whey) and 0.98 nm (corresponding to gelatin) to 1.24 nm for M hydrogel.

The addition of Cu determined the shift of the diffraction angle 2θ to higher values for diffraction peaks corresponding to helical structures, compared to sample M, revealing the increase in the interactions between chains, contributing to the decreasing of the diameter of the triple helix, and the distances between polypeptides chains. The increasing of the content of Cu for hydrogel samples determined major changes in the hydrogel structure leading to the decreasing of the crystallinity and destruction of helical structures, proved by the missing of the peak at 2θ ≈ 8° for samples III and IV. In the case of sample IV, the peak around 2θ ≈ 20° is missing, and a wide peak centered at 2θ ≈ 38° can be noticed. The diffraction peak at 2θ ≈ 36.91° is related to the distance between amino acid residues along the helix [52,53] exhibiting values of about 0.24 nm.

The values obtained for the distance between turns of helical structures and the distance between chains calculated from diffraction patterns are presented in Table 3.

Other small sharp peaks appears in the diffraction patterns of sample II at 9.15°, indicating the tendency of formation of new crystalline structures into the structure of hydrogels [40].

Other authors also observed that the interaction of divalent cations, such as Ca^2+^ with negatively charged carboxylic groups of adjacent protein chains, led to the formation of less ordered networks [14,54].

The decrease in crystallinity of gelatin-based materials following to the addition of tannin was observed by Pena et al. [40], due to the interactions between tannin and gelatin or due to the interactions between poly(vinyl alcohol) (PVA) and gelatin in blend hydrogel [2].

The research results of Kang et al. [32] showed that in the case of composite hydrogel based on whey and carbon nanotubes or carbon nano-onions the secondary structure of whey suffered minor changes due to the interactions with carbon based materials; the diffraction peak around 2θ ≈ 20° is still present in the diffraction patterns of composite materials.

As can be seen above, the interaction of proteins from hydrogels with both ionic and covalent compounds could lead to the denaturation of the proteins’ secondary structures and the decreasing of the diffraction peaks characteristic to triple helix structures. In the case of TiO_2_/whey protein isolate nanocomposite films [55], the XRD patterns revealed only changes related to the presence of the oxidic phase, proving that the secondary structure of whey suffered minor changes. One can conclude that the renaturation of the secondary structure of proteins during hydrogel formation can be inhibited by strong interactions with divalent metals with the formation of intermolecular ionic bonds between neighboring chains or covalent compounds that interact with the chains of proteins.

### 3.3. Fourier Transform Infrared Spectrometry (FTIR)

A FTIR analysis of the whey and gelatin-based hydrogels with copper content was employed to characterize the changes induced by the incorporation of CuSO_4_ × 5H_2_O into the hydrogels.

**Figure 8:**
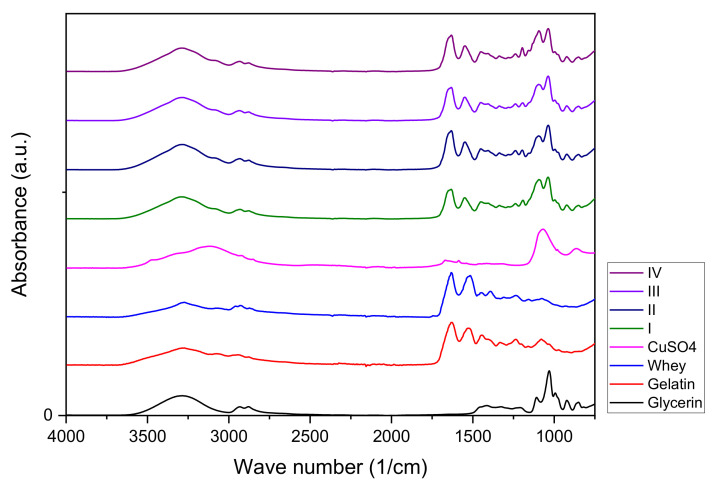
IR spectra of CuSO_4_ × 5H_2_O, whey, glycerin, and gelatin and hydrogel samples with different amounts of copper.

The IR spectra of the hydrogel, CuSO_4_ × 5H_2_O, whey, glycerin, and gelatin are shown in Figure 8. As can be seen, the spectrum of all samples exhibits characteristic bands for amide II and III (except the spectra of CuSO_4_ and glycerol), at wavenumbers 850–1600 cm^−1^ (CH and NH vibrations), amide I at wavenumber 1600–1700 cm^−1^ (C=O and NH vibrations and H bonds), amide B at wave number around 2920–2950 cm^−1^ (NH vibration and CH_2_ asymmetrical stretch) and amide A at wave number 3100–3300 cm^−1^ (NH vibration and H bonds). Furthermore, interactions between the OH group of glycerol and gelatin conduct to the band were noticed between 1030–1037 cm^−1^ [22].

The interaction between gelatin and whey and the presence of water from the hydrogels was conducted to the shift of Amide A bands to higher wavenumbers, from around 3273 cm^−1^ (corresponding to whey and gelatin) to around 3290 cm^−1^. In the case of amide I band (around 1632 cm^−1^), the interactions between whey, gelatin, and copper ions determined a shift of absorption bands towards higher wavenumbers for all hydrogels samples. The changes in the absorption bands can be also explained by the interactions between proteins and glycerol conducting in the formation of new stronger hydrogen bonds C=O---H-O (involving O-H groups of glycerol) then the bonds C=O---H-N from the proteins. Important changes can be noticed in the case of amide II absorption bands that vibrate at higher wavenumbers for all hydrogels (around 1550 cm^−1^), than the vibration of gelatin (1527 cm^−1^) or whey (1516 cm^−1^).

The main vibration bands of hydrogels samples are presented in Table 4.

Visible shifts of the amide III bands are observed from wavelengths of 1080.02 cm^−1^ (gelatin) and 1076.17 cm^−1^ (whey) to higher wavelengths numbers of approximately 1093 cm^−1^ (I, IV) and 1095 cm^−1^ (II, III), which can be attributed to changes in the secondary structure of proteins due to the addition of copper [6].

The FT-IR spectra of whey-based hydrogels with copper oxide nanoparticles (CuO NPs) [21] demonstrate good compatibility between the two components, as can be seen in our FT-IR spectra too.

Comparing the data obtained by IR spectroscopy and the one obtained by XRD, one can conclude that, at least in the case of protein-based hydrogels, XRD showed clear changes in the diffraction patterns of hydrogels as a function of copper concentration, while in the case of IR absorption bands, the changes are more subtle.

Rajabi et al. [14] hypothesize that the functional groups -COO^-^ of gellan gum and Cl^-^ ions in CaCl_2_ reduce the interactions between protein chains either by increasing electrostatic repulsions between groups with the same charge or by electrostatic attractions between positive and negative groups in the secondary structure of hydrogel network. The shift of the absorption peak of whey located at the wave number 1638 cm^−1^ (β-sheet structure) to 1649 cm^−1^, determined by the vibration of bound in C=O groups in the hydrogel, shows the formation of randomly coiled structures. Ca^2+^ ions can also stimulate the denaturation of proteins, especially β-Lg on hot gelation, illustrated in IR spectra by the shift of the absorption peak of whey hydrogel from 1449 cm^−1^ to 1447 cm^−1^, as was measured for whey hydrogel containing Ca^2+^. This was explained by the deformation of chemically bound N-H groups and hydrogen bonds in the secondary structure of proteins.

### 3.4. UV-VIS Spectroscopy

**Figure 9:**
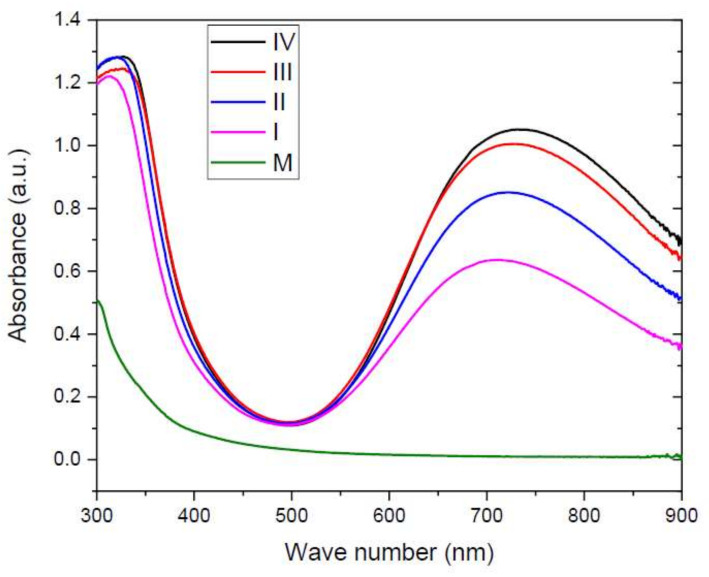
UV-VIS absorption spectra of hydrogel samples (M, I, II, III, IV).

After drying, the optical properties of the hydrogel samples, in the visible and near-infrared region of spectra (400–1000 nm), were determined. Protein-based hydrogels have good UV barrier properties due to the content of aromatic amino acids which can absorb UV light [22]. Figure 9 presents the UV-VIS spectra of the hydrogels.

The higher the concentration of copper sulphate, the higher the peak around 700 nm, and the peak around 350 nm notifies the presence of CuSO_4_. In the control sample case, the absorbance is due exclusively to the hydrogel proteins, which absorb in the UV domain. Hemalatha et al. [56] studied the influence of copper content on optical properties of polyvinyl alcohol/polyvinyl pyrrolidone polymer composite films doped with different amount of cupric sulphate (CuSO_4_), revealing that the transmission decreases with the increase in concentration of CuSO_4_, due to the formation of intermolecular bonding involving Cu^2+^ ions.

Arfat et. all [22] have found similar results in their study, wherein a gelatin-based hydrogel loaded with Ag-Cu NPs will show an absorbance the more intense the higher the Ag-Cu NPs content.

### 3.5. NMR Relaxometry

**Figure 10:**
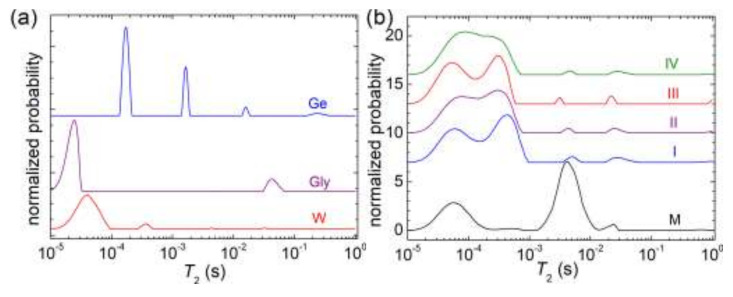
The ^1^H NMR *T*_2_-distributions measured for (**a**) raw materials: Whey (red), Glycerin (plume), Ge (blue), and (**b**) Whey: Gel with different content of Cu.

NMR relaxometry combined with inverse Laplace transform analysis is an advanced method that allows us to evaluate the state of water, from a dynamical point of view for our samples [57]. Figure 10a presents the *T*_2_-distributions measured for the raw materials such as whey, glycerin, and gelatin. One can observe that for whey (W) and glycerin (Gly), the largest amount of ^1^H is found in extremely rigid components (see the large peak located at small *T*_2_-values, below 0.1 ms). Whey presents small peaks, which can be associated with ^1^H in rigid components (the peak located at about 367 µs), with less mobile (the peak located at about 3.33 ms,) and medium mobile (the peak located at about 32.4 ms) components. Glycerin presents another peak in the medium mobile domain (located at about 42.5 ms) with a significant contribution. Approximately in the same range of *T_2_*, the *T_2_-*distributions measured for samples of starch/glycerol/water in 68/17/15 mass % ratio are reported in ref. [58]. The *T*_2_-distribution measured for the gelatin samples looks completely different than the *T*_2_-distribution measured for glycerin. This one (top distribution in Figure 10a) presents three narrow peaks, located in the rigid domain (with the largest amount of ^1^H) characterized by *T*_2_-values of about 170–203 µs, the less mobile domain characterized by *T*_2_-values of about 1.3–1.65 ms and with the smallest amount of ^1^H in the domain of medium mobility characterized by *T*_2_-values of about 15.8–19.2 ms. Additionally, the gelatin sample (Ge) presents a small and wide peak in the domain of mobile protons located at about 237 ms.

The *T*_2_-distributions measured for the reference sample (M) and whey (gel and Cu in different amounts) are presented in Figure 10b. One can observe that for the reference sample (M), the largest amount of ^1^H is found in the domain of less mobile protons. Additionally, a rigid component containing a significant amount of ^1^H is observed at low *T*_2_-values and matches whey’s extremely rigid component (see the red distribution in Figure 10a). Additionally, small peaks are observed in the rigid and medium domains. The samples containing Cu in different amount present similarities of the measured *T*_2_-distributions and are completely different compared to the *T*_2_-distribution measured for the reference sample (without Cu). With a high degree of probability, one can assume that the main effect of the presence of Cu in our samples is to take ^1^H located into a less mobile component (*T*_2_-values of the order milliseconds) and transferred it to rigid component (*T*_2_-values or the order of hundreds of µs). This is a clear fact that the Cu induces the cross-linking of the prepared hydrogels. The *T*_2_-distributions measured for the hydrogels containing Cu present the majority of protons (the nucleus measured by ^1^H NMR) in extremely rigid and rigid components observed as a doublet located at low *T*_2_-values. The mixing of whey with glycerin results in a dynamical component characterized by a medium rigidity (that of whey) located at *T*_2_-values of the order of tens of microseconds (51–89 µs), while the second one is located at *T*_2_-values of the order of hundreds of microseconds (220–434 µs) and is mostly due to the presence of Cu and presents an integral area dependent on the Cu content. Additionally, to these peaks, one can observe a rest of ^1^H located at *T*_2_-values of the order of 3.1–5.1 ms and which can be associated with less mobile components, and another rest of ^1^H located at *T*_2_-values of the order of tens of milliseconds (22.1–28.8 ms) and associated with medium mobile components. Both of the last components can also be found in the reference samples (without Cu) but seems that the Cu prefers with predilection to be associated with the ^1^H reservoir which is found in the less mobile component to lead to the cross-linking of the polymer chains.

**Figure 11:**
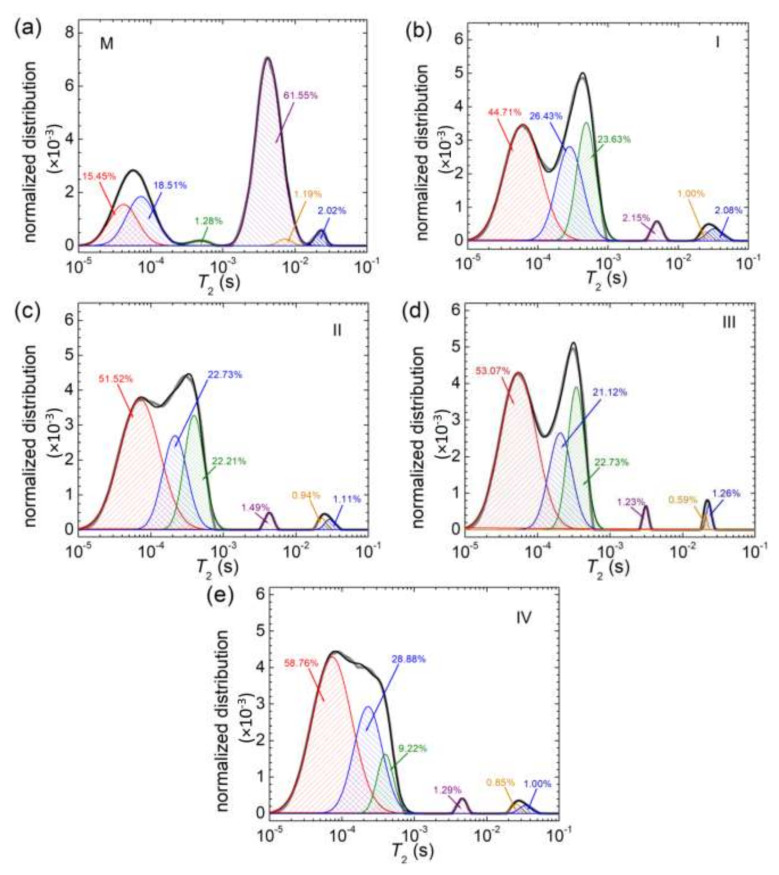
The deconvolution with six dynamics components of *T*_2_-distributions measured for (**a**) M; (**b**) I; (**c**) II; (**d**) III and (**e**) IV.

For a better understanding of the mechanism of copper mediated cross-linking of the polymer network, the deconvolutions of *T*_2_-distribution were performed [43] and some results are presented in Figure 11. Thus, in Figure 11a, the deconvolution of the *T*_2_-distribution measured for the reference sample, M (without Cu) is presented. Several (six) components could be identified. Two of them compose the peak located at low *T*_2_-values and are associated with the extremely rigid components of hydrogels. Another two peaks seem to compose the peak located in the region of less mobile components (*T*_2_-values of the order of milliseconds). On the contrary, for the samples containing Cu, the peak located at the smallest *T*_2_-values (tens of microseconds) seems to contain only a single component and one can identify two components composing the peak located at hundreds of microseconds. Then, in one process, in the cross-linking process, one can assume that the Cu will use the components with less mobility, and also components with extreme rigid mobility, to increase the mobility of extremely rigid components and to decrease the mobility of the less mobile components, creating the new rigid component. In another process, one can observe that the percentage of ^1^H in extremely rigid components measured for the reference sample, without Cu, is only 33.96 (%) located in two components, a percentage which increased up to 44.71 for sample I, with the smallest amount of Cu. This means that Cu is involved in the increases in the ^1^H amount of extremely rigid dynamic components of hydrogels with Cu.

### 3.6. Antimicrobial Activity Evaluation

It was observed that in all strains control sample (M) showed no inhibition, but the other samples tested varied in the size of the inhibition diameter depending on the microbial strain tested and copper sulphate concentration (Table 5).

In the bacterial strain, *Porphyromonas gingivalis* an inhibition was observed in all samples tested with differences depending on the type of sample (6.5–11.5 mm). The lowest inhibition value was recorded in samples I (6.5 mm) and II (8 mm). In general, the inhibition of this bacterium was slightly lower compared to the other bacterial strains studied.

In the bacterial strain, *Enterococcus faecalis* a slight inhibition was observed in all four samples (I–IV). The inhibition recorded in this strain was almost identical to that recorded in the *P. gingivalis* strain.

In the bacterial strain *Streptococcus mutans,* higher inhibition was observed in samples III (15 mm) and IV (15.5 mm) compared to the other two samples.

In the *Escherichia coli* strain, a rather high inhibition was observed compared to the other strains tested, especially in sample III (20 mm) and IV (25 mm).

In the bacterial strain *Staphylococcus aureus* an inhibition was observed in all four samples (I–IV) tested, but this was lower. In this case, too, sample III and IV showed a slightly higher inhibition compared to the other samples.

**Figure 12:**
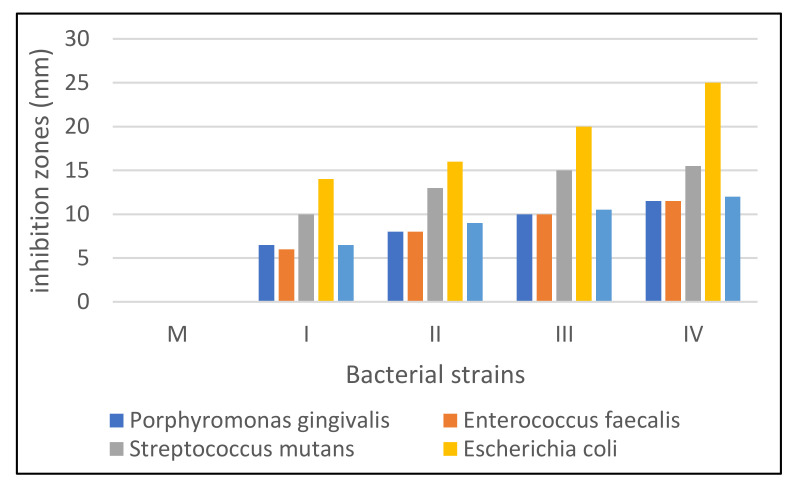
Bacterial sensitivity to tested hydrogels.

Graphically representing the diameters of inhibition zones in the strains studied, it can be seen that the highest inhibition was *E. coli*, followed by *S. mutans*. The lowest inhibitions were recorded in *S. aureus* (Figure 12).

Based on these results, it can be stated that copper-enriched gels have different antimicrobial activity depending on the composition of hydrogel (the concentration of copper sulphate) and depending on the bacterial strain tested. These qualities make them good candidates for antibacterial wound dressings, cosmetic, and food packages. Further investigations may bring more insight into the antimicrobial mechanism of copper in biocomposite and hydrogels.

The antimicrobial activity of copper compounds, proportional to the Cu^2+^ ion concentration, has been proven in food and medical research. Arfat and col. [22] have investigated bionanocomposite films based on fish skin gelatin (FSG) and bimetallic Ag-Cu nanoparticles (Ag-Cu NPs), which was found to have an elevated antibacterial activity against both *Salmonella enterica sv Typhimurium* and *Listeria monocytogenes* bacteria with time incubation.

Nanocomposites based on whey protein isolate containing copper oxide nanoparticles (CuO NPs) have demonstrated synergistic antimicrobial activity with coconut essential oil and paprika extract with the highest results against *Staphylococcus aureus* and *Escherichia coli* [21].

A copper (II) sulphate cross-linked alginate hydrogel films [40] demonstrate antibacterial activity against *Escherichia coli, Staphylococcus aureus,* methicillin-resistant *Staphylococcus aureus, Staphylococcus epidermidis, and Streptococcus pyogenes.* This paper [59] highlights also the effect on pro-thrombotic coagulation and platelet activation of copper ions.

Another combination between copper ions and bio-films hydrogels has been studied by Oun and col. [60]. Their work shows similar results in the antimicrobial activity of copper oxide nanoparticles (CuO NPs), incorporated in carrageenan-based hydrogels, against food-borne pathogenic bacteria: *Escherichia coli* and *Listeria monocytogenes*.

## 4. Conclusions

The present study describes a hydrogel based on whey and gelatin, plasticized with glycerin, and cross-linked with different amounts of CuSO_4_ to improve hydration properties and stability in liquid systems based on water. Swelling tests revealed that the new hydrogels are stable up to a week in distilled water. We established the minimum concentration of copper sulfate in the samples (0.55%) for obtaining long lasting hydrogels.

The swelling process followed the Fickian model up to 60% of the hydration capacity, but over the full hydration interval, the swelling demonstrated an atypical diffusion following Schott’s equation.

X-ray diffraction revealed that the addition of copper ions determined changes in the secondary structure of proteins, even at low concentrations, FT-IR spectroscopy confirmed changes in the secondary structure of hydrogels following the addition of copper and illustrated changes in the structure of hydrogels.

By revealing the hydrogel network dynamics, the ^1^H NMR relaxometry measurements clearly indicate that Cu is an efficient cross-linking agent. The results obtained in this work indicated the antimicrobial potential of whey-based composite hydrogels cross-linked with copper compounds, therefore encouraging further study of this type of hydrogel in the food packaging field.

## Figures and Tables

**Table 1:** The composition of hydrogel samples.

Sample	M	I	II	III	IV
Copper sulphate concentration (%) *	0	0.39	0.55	0.70	0.86

* The concentration of CuSO_4_ × 5H_2_O in the wet sample before drying.

**Table 2:** The values of the rate constant (*K*) and the swelling degree (*SWe*) calculated based on Schott’s equation and experimentally determined.

Code Sample	Slope	*SWe* (Calculated) (%)	*SWe* Experimental (%)	Intercept	*K*×10^5^ (1/min) ×10^5^
I	0.00229	434.78	383.55	0.1244	4.25
II	0.00131	769.23	772.03	0.1644	1.03
III	0.00126	769.23	783.20	0.1331	1.27
IV	0.00125	769.23	745.63	0.1661	1.02
M	0.00191	526.31	504.39	0.2653	1.36

**Table 3:** The structural characteristics calculated from diffraction patterns for gelatin (G), whey (W), whey–gelatin (M), and whey–gelatin hydrogels crosslinked with copper (I, II, III, IV).

Sample	2θ (Degree)	Distance between Turns (nm)	2θ (Degree)	Distance between Chains (nm)
G	9.89	0.894	20.21	0.439
W	9.02	0.979	19.85	0.447
M	7.10	1.244	20.37	0.436
I	7.45	1.186	20.39	0.435
II	7.89	1.186	20.44	0.434
III	-	-	21.07	0.421
IV	-	-	36.91	0.243

**Table 4:** The main vibration bands of hydrogels and their components.

Glycerin	Gelatin	Whey	I	II	III	IV	Attributions
(cm^−1^)							
3285.58	3272.82	3272.4	3291.67	3290.14	3291.20	3291.41	Amide A, tensile vibrations NH [14,21,41], hydrogen bonds [14,54]
-	1630.62	1631.72	1633.51	1631.75	1633.32	1632.03	Amide I, C=O and NH vibrations [14,21,22,41], H bonds coupled with COO^−^ [14,22,54]
-	1527.47	1515.90	1550.56	1548.66	1549.63	1548.64	Amide II, CN vibrations/stretching, NH bending [14,22]
1413.81	1445.43	1446.41	1450.33	1450.35	1451.20	1450.27	Amide II, CN vibrations, NH bending [21]
-	1080.02	1076.17	1093.52	1095.46	1095.43	1095.73	Amide III CN and NH vibrations [6,21]

**Table 5:** Diameters of inhibition zones (mm) for tested hydrogel samples.

Bacterial Strain	Sample Code				
	M	I	II	III	IV
*Porphyromonas gingivalis*	0	6.5	8	10	11.5
*Enterococcus faecalis*	0	6	8	10	11.5
*Streptococcus mutans*	0	10	13	15	15.5
*Escherichia coli*	0	14	16	20	25
*Staphylococcus aureus*	0	6.5	9	10.5	12

## Data Availability

All the data is available within the manuscript.
